# Design, Synthesis, and Antitumor Activity of Isoliquiritigenin Amino Acid Ester Derivatives

**DOI:** 10.3390/molecules29112641

**Published:** 2024-06-03

**Authors:** Chi Liu, Xinyue Liu, Qing Ma, Fengyan Su, Enbo Cai

**Affiliations:** College of Chinese Medicinal Material, Jilin Agricultural University, 2888 Xincheng Street, Changchun 130118, China; liuchi0222@163.com (C.L.); lxy533zai@163.com (X.L.); mq15141834269@163.com (Q.M.)

**Keywords:** isoliquiritigenin derivatives, cervical cancer, antitumor, PI3K/Akt/mTOR signaling pathway

## Abstract

Isoliquiritigenin (ISL) is a chalcone that has shown great potential in the treatment of cancer. However, its relatively weak activity and low water solubility limit its clinical application. In this study, we designed and synthesized 21 amino acid ester derivatives of ISL and characterized the compounds using ^1^H NMR and ^13^C NMR. Among them, compound **9** (IC_50_ = 14.36 μM) had a better inhibitory effect on human cervical cancer (Hela) than ISL (IC_50_ = 126.5 μM), and it was superior to the positive drug 5-FU (IC_50_ = 33.59 μM). The mechanism of the action experiment showed that compound **9** could induce Hela cell apoptosis and autophagy through the PI3K/Akt/mTOR pathway.

## 1. Introduction

Cancer is a major disease that threatens human health. According to the 2020 study report published by the Agency for Cancer study of the World Health Organization, there are roughly 19.2928 million new cases of cancer worldwide, with 9.9581 million new cancer deaths [1]. The report also projects a modest annual increase in the number of new cases. Owing to the intricate etiology and challenging aspects of cancer, the three primary therapeutic modalities still used in cancer care are surgery, radiation therapy, and chemical medication therapy [2].Despite the fact that all three treatments can increase patient lifespan, some cancers cannot be surgically removed, radiation and chemotherapy can cause severe side effects, and chemotherapy can cause drug resistance [3,4]. A medication created in accordance with the principles of traditional Chinese medicine is known as traditional Chinese medicine. Because of its distinct anti-tumor action and minimally hazardous side effects, it is frequently employed in the treatment of malignancies. About half of the 80 different types of anti-tumor medications currently in clinical use worldwide are derived either directly or indirectly from plants. Commonly used plant-based medications include vinblastine [5], paclitaxel [6], artemisinin [7], betulinic acid [8], berberine [9], ginsenosides [10], etc.

One chalcone component that can be separated from the leguminous plant *Glycyrrhiza uralensis* was isoliquiritigenin [11]. It has a wide range of pharmacological properties, including anti-inflammatory [12], anti-tumor [12], anti-angiogenesis [13], antibacterial [14], and anti-diabetic activities [15]. For instance, it possesses many modes of action and exhibits some inhibitory effect on human cancer cells, including those of the liver [16], breast [17], prostate [18], and cervical regions [19]. By preventing the cell cycle, triggering tumor autophagy, preventing tumor cell spread, and inducing apoptosis, among other mechanisms, it can cause tumor cells to die [20]. However, the application of isoliquiritigenin is still limited by its poor solubility and low anti-tumor activity.

Amino acids are the fundamental units of proteins and the main substances that maintain biological life activities. The chemical structure of an amino acid includes an amino group (-NH_2_) a carboxyl group (-COOH), and a unique carbon side chain connecting the amino and carboxyl groups. Amino acid structures are simple and diverse, with significant anti-tumor activity, making them easy to use in drug synthesis and structural modification. According to reports, after combination with an amino acid, the solubility of the compound is enhanced, the selectivity towards tumor cells is improved, and the toxicity to normal cells is reduced [21]. In the preliminary research results of the research group, the anti-tumor activity was significantly enhanced by the introduction of amino acids separately, such as Arctigenin [22], α-mangostin [23], and podophyllotoxin [24]. A common chalcone structure, isoliquiritigenin, is joined to the A and B rings by cross-conjugation with propylene ketone. Three atoms with large rotational degrees of freedom are present in the associated bridge bonds. Consequently, isoliquiritigenin’s binding to receptors has a wide geographical distribution. Ref. [25] verified that isoliquiritigenin’s anti-tumor action requires the presence of hydroxyl groups as functional groups. According to research [19], the best locations for chalcone compounds to substitute active groups are at positions 2′and 4′ of the A ring and 4 of the B ring. Structure–activity relationship analysis demonstrated that, following chlorination modification at positions 2′ and 4′ of the A-ring, the modified chlorination at these locations had considerably greater activity on Hela and SiHa cells and less toxicity on normal CHO cells than at positions 3′, 4′, 2′, and 5′.

Therefore, this study designed and synthesized a series of amino acid derivatives of isoliquiritigenin, screened out isoliquiritigenin derivatives with stronger anti-tumor activity, and conducted preliminary discussions on the structure–activity relationship and apoptosis mechanism, providing theoretical reference for the further development and utilization of isoliquiritigenin.

## 2. Results and Discussion

### 2.1. Chemistry

The structures of all synthesized target derivatives were characterized by ^1^H NMR, ^13^C NMR, and HRMS. The characterization data were consistent with the predicted structures.

**Scheme 1:**
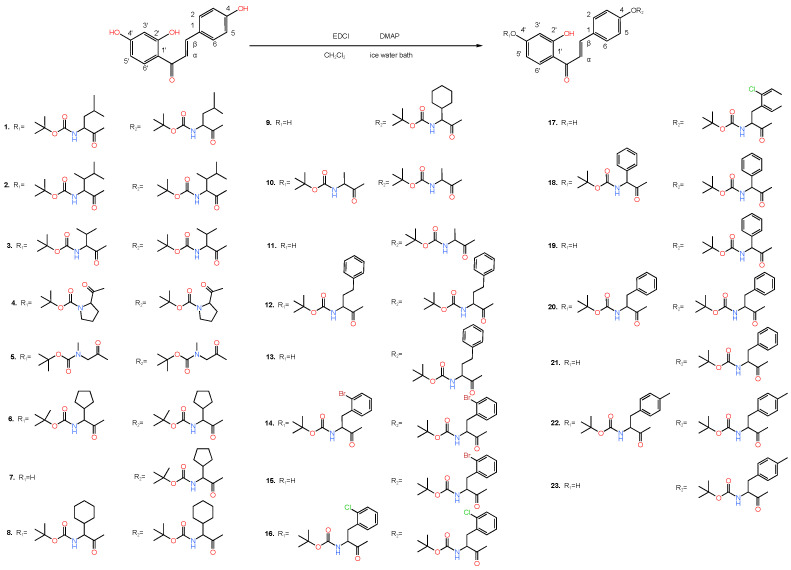
Synthetic route of target compounds **1**–**23**.

According to Scheme 1, using isoliquiritigenin as raw material, dichloromethane (CH_2_Cl_2_) as reaction solvent, 4-dimethylaminopyridine (DMAP) as a catalyst, and 1-ethyl-(3-dimethylaminopropyl) carbodiimide hydrochloride (EDC · HCl) as a decarboxylation agent under ice-water bath reaction conditions, various amino acids with different structures were selected for chemical synthesis. Finally, 23 target derivatives were separated by silica gel column chromatography (**1**–**23**).

### 2.2. Toxicity of Compounds on Different Human Tumor Cell Lines

The anti-tumor activity of all target compounds **1**–**23** was evaluated in vitro by an MTT assay against Bel-7402, A549, Hela, MCF-7 and PC-3M cell lines, with 5-FU as the positive control. Their inhibition rates and IC_50_ values are listed in Table 1.

The following structure–activity relationship pattern was obtained by assessing the anti-tumor activity of the aforementioned compounds: (1) The structurally modified derivatives had a lower overall inhibitory action on tumor cells than the mother nucleus isoliquiritigenin when the C4′-OH of the A ring and the C4-OH of the B ring of isoliquiritigenin were esterified concurrently; and (2) the structurally modified derivatives exhibited a greater overall inhibitory action on tumor cells than isoliquiritigenin, the parent nucleus, when C4-OH on the B ring was esterified alone. Four of them had far greater inhibitory effects against tumor cells when modified with amino acids that had cycloalkyl groups or halogen atoms. Specifically, compound **21** had an IC_50_ value of 66.5 for Hela cells and 87.5 μM for PC-3M cells. This is because compound **21** esterifies phenylalanine on the B-ring alone by C4-OH without adding halogen atoms. However, the IC_50_ values of compound **15** and compound **17** on Hela dropped to 27.36 and 30.4 μM, respectively, upon the introduction of halogen atoms Br and Cl. The PC-3M’s IC_50_ values were lowered by compound **15** and compound **17** to 30.2 μM and 37.4 μM, respectively. Furthermore, the inhibitory activity against PC-3M cells was significantly increased by adding cyclic alkyl amino acids, specifically compound **7** and compound **9**, with compound **9** exhibiting the largest change in activity. For Hela cell activity (IC_50_ = 14.36 μM), isoliquiritigenin (IC_50_ = 126.5 μM) increased by about 10 times and was better than the positive drug 5-Fu (IC_50_ = 33.59 μM).

To further confirm the toxicity of compound **9** to normal cells, we used the MTT method to determine the cytotoxicity of human normal cervical epithelial cells (HUCEC), and the results showed that compound **9** had cytotoxicity (IC_50_ > 200 μM). Therefore, compound **9** can serve as a specific mechanism for further research.

### 2.3. Compound ***9*** Inhibits Hela Cell Migration

**Figure 1:**
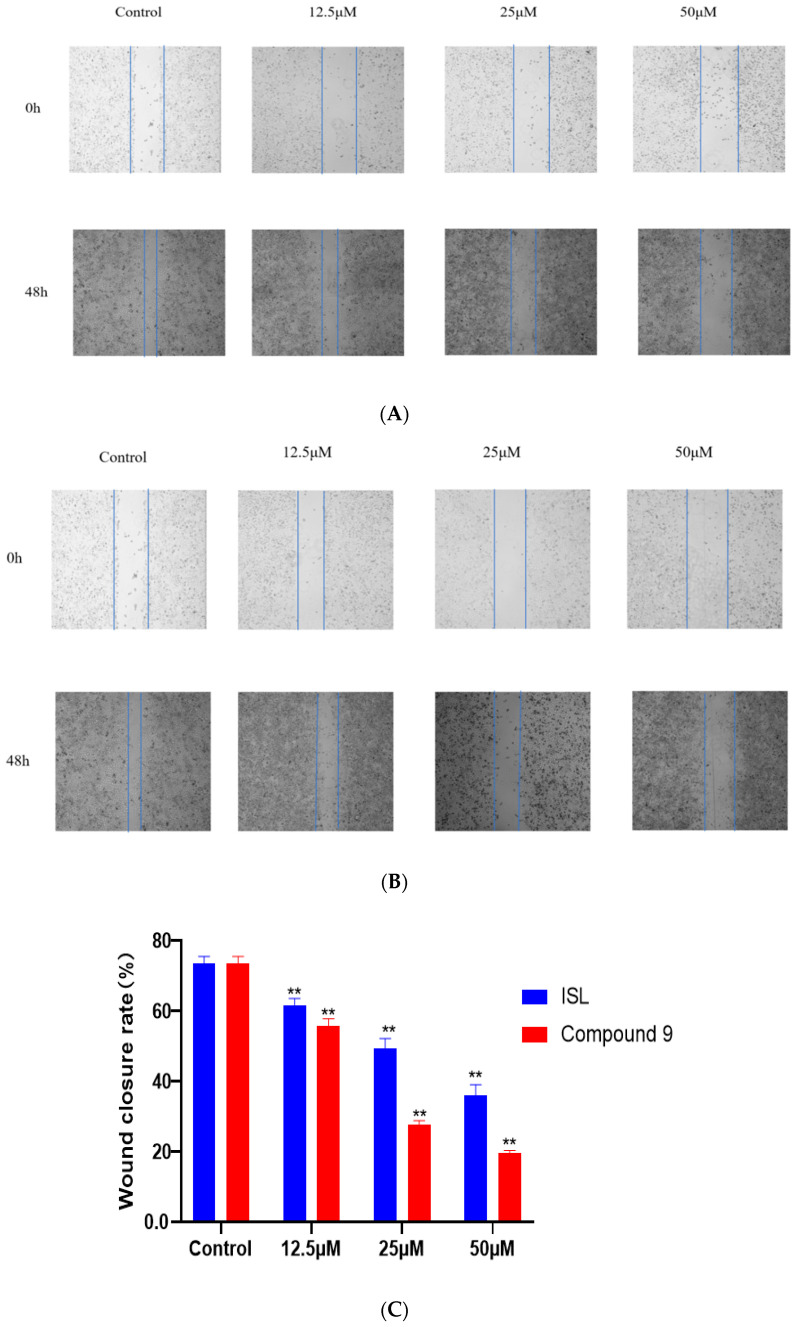
(**A**) ISL inhibits the migration of Hela cells under microscopy image of the degree of healing of cell scratches. (**B**) Compound **9** inhibits the migration of Hela cells under microscopy image of the degree of healing of cell scratches. (**C**) Scratch healing rate compared to the control group, ** *p* < 0.01.

Inhibiting tumor cell migration is a therapeutic approach for malignancies, as it is a necessary condition for invasion and metastasis. In order to verify the effects of ISL and compound **9** on the in vitro migration ability of Hela cells, the scratch test results showed in Figure 1, compared with the control group, compounds ISL and **9** could inhibit the migration of Hela cells. Compared with the ISL group, the scratch healing degree of the compound **9** group was relatively more significant, and the scratch healing degree significantly decreased with the increase in drug concentration.

### 2.4. Compound ***9*** Inhibits Hela Cell Proliferation

**Figure 2:**
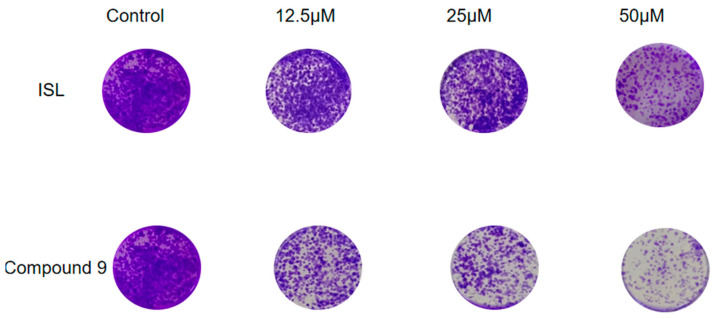
ISL and compound **9** inhibit the proliferation of Hela cells.

In order to verify the proliferative and killing effect of compound **9** on cervical cancer cells, this study used plate clone formation experiments to detect the effects of compound **9** and ISL on Hela cell clone proliferation ability. The results showed in Figure 2, compared with the control group, both compound ISL and compound **9** could inhibit Hela cell proliferation. Compared with the ISL group, compound **9** group had relatively fewer colonies, and the number of colonies significantly decreased with increasing drug concentration.

### 2.5. Compound ***9*** Affects Hela Cell Morphology

**Figure 3:**
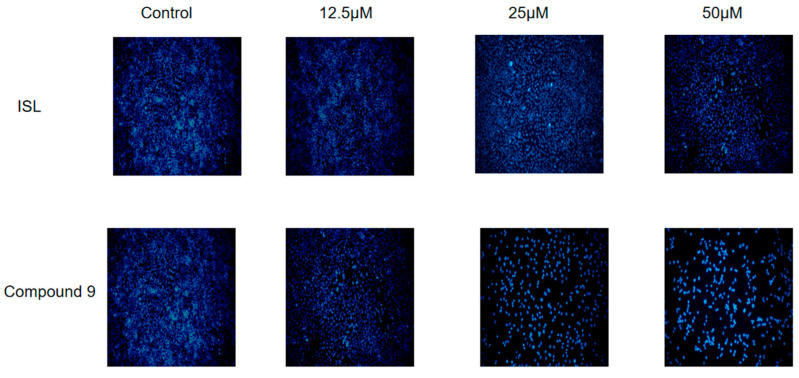
DAPI staining was used to detect the effects of ISL and compound **9** on the morphology of Hela cells.

As shown in Figure 3, compared with the control group, compounds ISL and **9** could both inhibit the apoptosis of Hela cells. The control group cells showed a good growth status, with spherical and intact nuclei. However, as the drug concentration increased, the number of cells in the treatment group decreased, and apoptosis began to occur, resulting in fragmented nucleus. The number of cells in compound **9** of 50 μM decreased sharply, and there were basically no complete nuclei present. Therefore, it can be concluded that this derivative can induce apoptosis of Hela cells and exhibits a certain dose-dependent effect.

### 2.6. Compound ***9*** Affects Hela Cell Apoptosis

**Figure 4:**
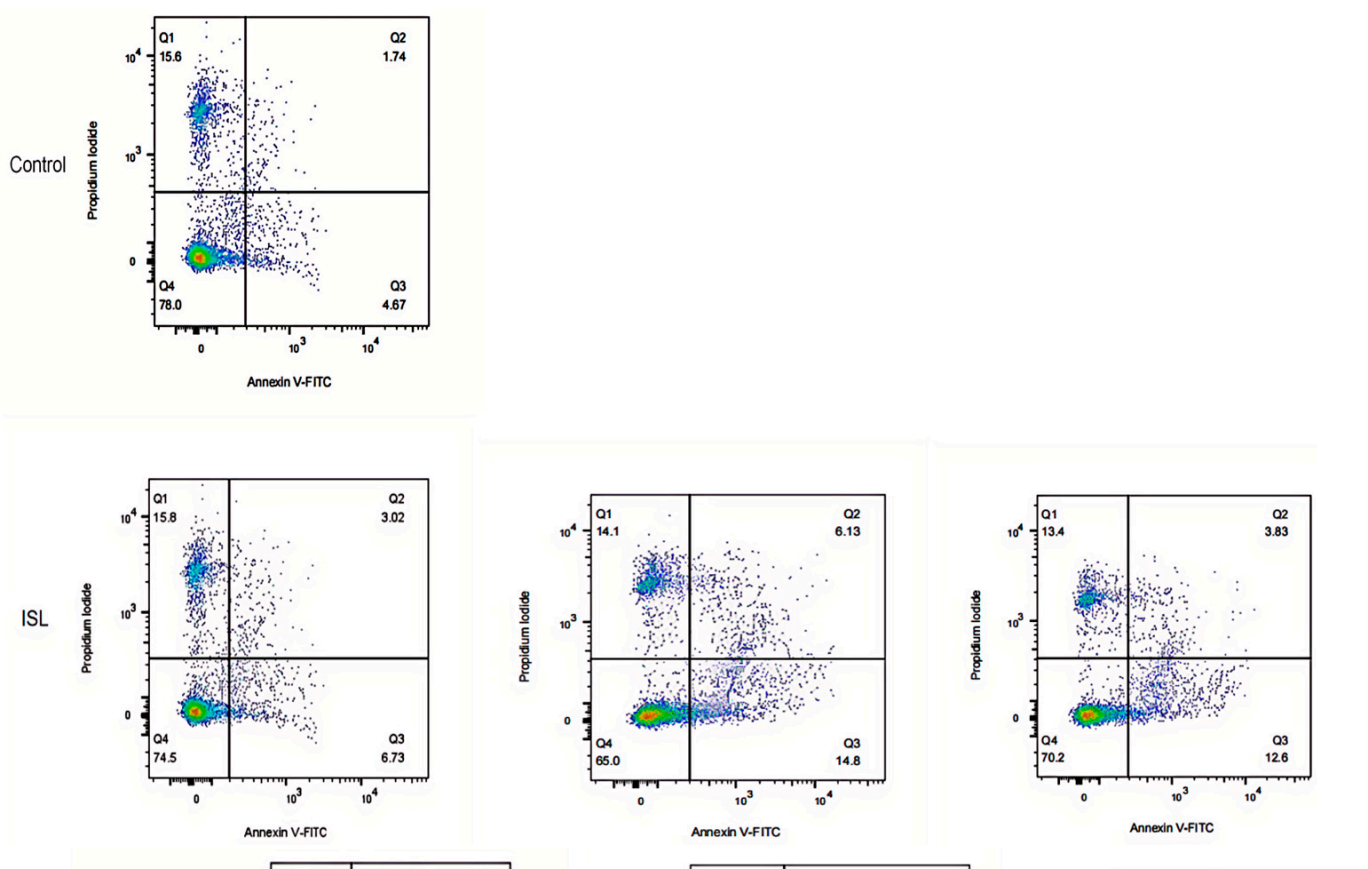

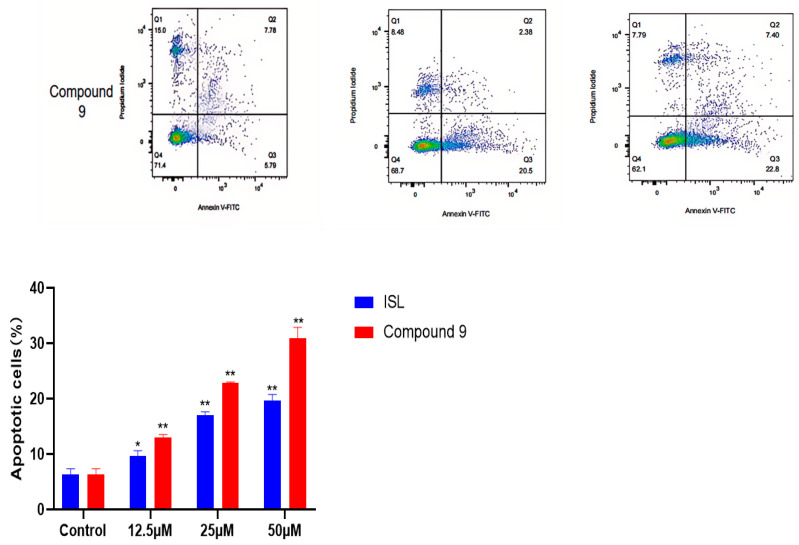
Annexin V-FITC/PI double-staining assay was used to investigate the effect of compound **9** on the apoptosis of Hela cells; compared to the control group, * *p* < 0.05, ** *p* < 0.01.

Quantitative analysis of apoptosis by Figure 4 showed that the apoptosis ratio of Hela cells in the control group was 6.41%, while the apoptosis ratios of Hela cells in the 12.5 μM, 25 μM, and 50 μM groups after 48 h of treatment were 13.57%, 22.88%, and 30.2%, respectively. The apoptotic proportions of Hela cells were 9.75%, 16.43%, and 20.93% after 48 h of treatment in the ISL group, which confirmed that compound **9** could induce apoptosis of Hela cells.

### 2.7. Anti-Tumor PI3K/AKT/mTOR Pathway Analysis of Compound ***9*** Based on Molecular Docking Technology

Molecular docking is a widely used computational tool in molecular recognition research whose purpose is to predict the binding pattern and binding affinity of a complex composed of two or more molecules of known structure [26]. At present, molecular docking has been widely used in the efficacy evaluation and mechanism of action of TCM, such as preliminary verification of targets, interpretation of the TCM mechanism of action, drug function localization, and identification of toxic components of TCM [27].

At present, studies have confirmed that the occurrence and development of cervical cancer are closely related to apoptosis and autophagy [28]. Autophagy is a catabolic pathway through which some cellular components are transported to lysosomes by autophagosomes [29]. Autophagy-related factors Beclin1, LC3, and p62, as means to assist in the diagnosis of cervical cancer lesions, are also the targets of a variety of anti-tumor drugs. The PI3K/AKT/mTOR signaling pathway related to autophagy also regulates a variety of cellular processes in the process of tumor occurrence, including tumor growth, tumor survival, tumor cell proliferation, tumor immunity, tumor metabolism, and tumor angiogenesis [30]. The activation of the PI3K/AKT/mTOR signaling pathway begins with the activation of growth factor receptors, cytokines, hormones, etc., on phosphatidylinoinosidine-3-kinase. PI3K enables PIP2 on the cell membrane to produce a second messenger, PIP3, which binds to the signaling proteins AKT and PDK1 containing the PH domain in the cell. AKT is translocated in the cell membrane and achieves catalytic activity, catalyzing its own phosphorylation at Serl24 and Thr450. PDKl can catalyze phosphorylation of the AKT protein at Thr308. AKT may also be fully activated by the phosphorylation of Ser473 by mTORC2 [31]. Activated AKT activates or inhibits a series of downstream substrates such as mTOR, Bad, Bcl-2, Caspase-3 and P70S6K through phosphorylation targeting, thereby regulating cell proliferation, differentiation, apoptosis, and migration.

**Figure 5:**
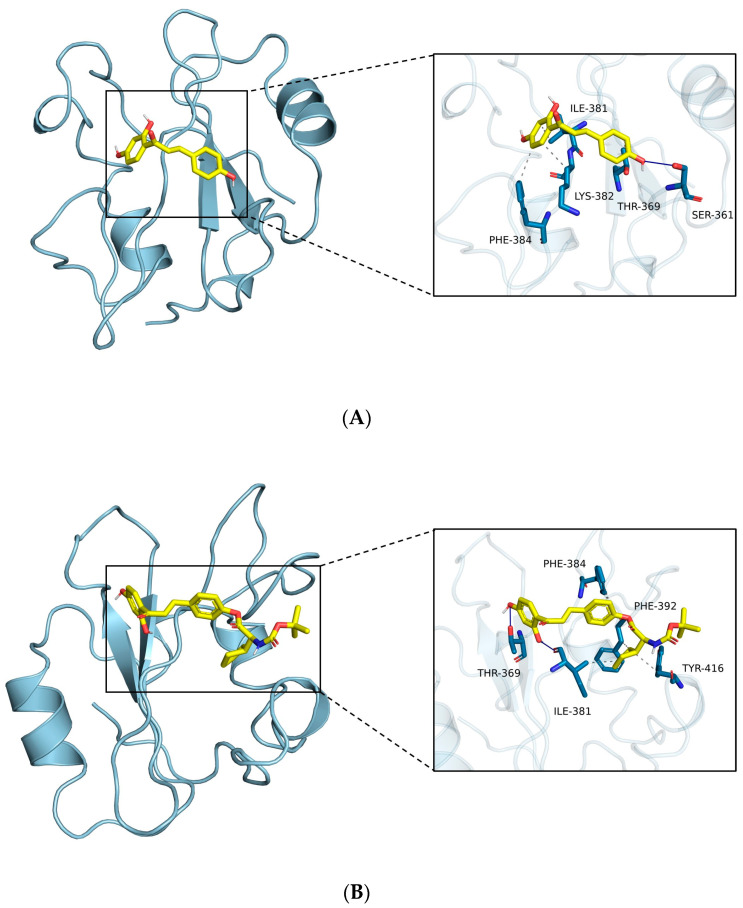

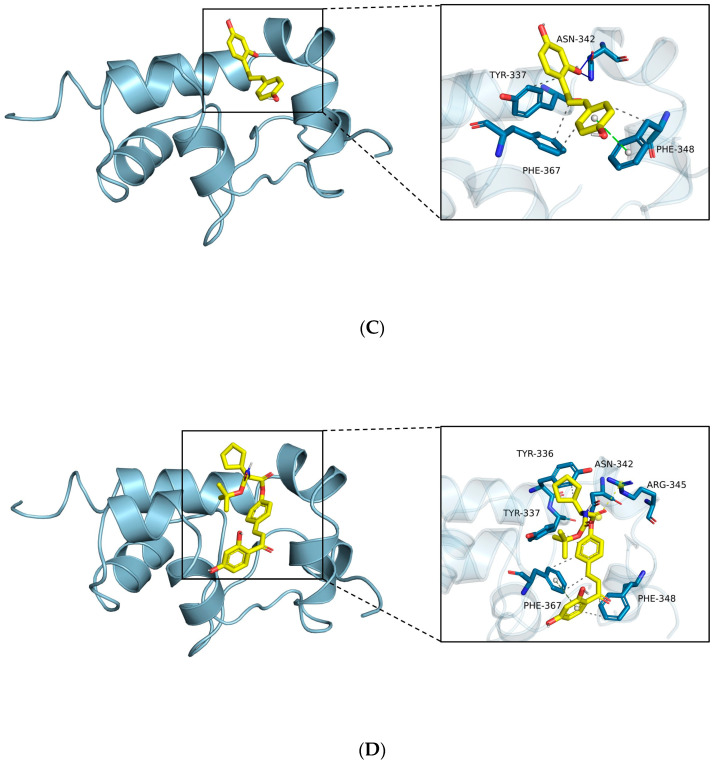
(**A**) Three-dimensional map of ISL-PI3K protein. (**B**) Three-dimensional map of compound **9**-PI3K protein. (**C**) Three-dimensional map of ISL-mTOR protein. (**D**) Three-dimensional map of compound **9**-mTOR protein.

In order to explore the binding ability of ISL and compound **9** to PI3K/Akt/mTOR pathway proteins, PI3K and mTOR were selected for docking analysis.As showed Figure 5, Auto Dock data showed that ISL formed hydrophobic interactions with the residues THR 369, ILE 381, LYS 382, and PHE 394 in the PI3K protein. Meanwhile, hydrogen bonding with the residue SER361 was measured to be 3.20 ngstrom. And the binding affinity was 8.1 kcal/mol. However, compound **9** formed hydrophobic interactions with the residues ILE381, PHE 384, PHE 392, and TYR 416 in the PI3K protein. Hydrogen bonding with the residue THR 369 was measured to be 2.54 ngstrom, and the residue ILE 381 was measured to be 4.06 ngstrom. And the binding affinity was 8.9 kcal/mol. Furthermore, ISL formed hydrophobic interactions with the residues TYR 337, PHE 348, and PHE 367 in the mTOR protein. Hydrogen bonding with the residue ASN 342 was measured to be 2.50 ngstrom. And the binding affinity was 6.9 kcal/mol. Nevertheless, compound **9** formed hydrophobic interactions with the residues TYR 336, TYR 337, PHE 348, and PHE 367 in the mTOR protein. Hydrogen bonding with the residue ASN 342 was measured to be 3.16 ngstrom. And the binding affinity was 7.6 kcal/mol. Thus, compound **9** can be further studied on the PI3K/Akt/mTOR pathway.

### 2.8. Real-Time Fluorescence Quantitative PCR Was Used to Detect PI3K/AKT/mTOR Pathway-Related Gene Expression

**Figure 6:**
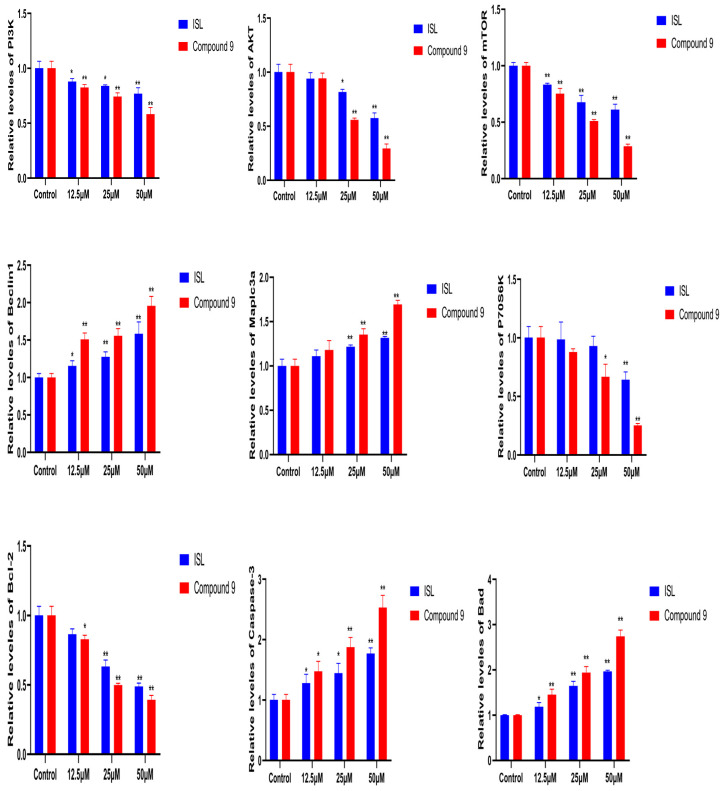
Effects of ISL and compound **9** on mRNA expression in Hela cells; compared to the control group, * *p* < 0.05, ** *p* < 0.01.

As shown in Figure 6, based on the prediction results of molecular docking, the star genes PI3K, AKT, and mTOR; the downstream genes P70S6K, Bad, Bcl-2, and Caspase-3; and autophagy-related genes Maplc3A and Beclin1 in the PI3K/AKT/mTOR pathway were detected in this experiment, and the results showed that, compared with the control group, PI3K, AKT, mTOR, and P70S6K, the mRNA levels of five genes of Bcl-2 showed a downward trend, and the mRNA levels of three genes, Bad, Maplc3A, and Beclin1, showed upward trends. In addition, compared with ISL, the changes in compound **9** groups were more significant.

## 3. Materials and Methods

### 3.1. Chemistry

Every chemical and solvent was above analytical purity. Purchased from commercial vendors, all reagents and solvents were utilized without additional purification. A QSTAR spectrometer was used to collect HR-ESI-MS data (FL, USA). Utilizing an American Varian NMR System 300 MHz, NMR spectroscopy was conducted in CDCl_3_ to determine chemical shift (δ) values, with solvent peaks or tetramethylsilane (0.00 ppm) serving as the internal reference. All compounds were separated using silica gel columns (Qingdao Ocean Chemical Group Co., Ltd., Qingdao, China).

### 3.2. Preparation of Isoliquiritigenin Derivatives

Isoliquiritigenin with a purity of 98% was used as a raw material (Chengdu Na Lithium Biotechnology Co., Ltd., Chengdu, China). As shown in the procedure in Scheme 1, isoliquiritigenin (1.0 mmol), different kinds of amino acids with Boc protection (3.0 mmol, 3.0 eq), 1-Ethyl-3-(3-dimethylaminopropyl)carbodiimide (3.0 mmol, 3.0 eq), and 4-Dimethylaminopyridine (1.0 mmol, 1.0 eq) were added to 20 mL of CH_2_Cl_2_ and reacted in an ice bath for 12–24 h. The reaction was detected by TLC. After the reaction was complete, the crude product was obtained by reducing the pressure concentration. Finally, target products were purified using petroleum ether (PE): acetone (AC).

**(*E*)-4-(3-(4-(((tert-butoxycarbonyl)leucyl)oxy)-2-hydroxyphenyl)-3-oxoprop-1-en-1-yl)phenyl (tert-butoxycarbonyl)leucinate (Compound 1):** Silica gel chromatography (PE/AC = 6:1) separated to give a yellow, viscous solid, yield 78.4%. ^1^H NMR (300 MHz, CDCl_3_) δ 13.00(s, 1H), 7.94(d, J = 4.5 Hz, 1H), 7.90(d, J = 10.8 Hz, 1H), 7.70(s, 1H), 7.67(s, 1H), 7.54(d, J = 15.6 Hz, 1H), 7.21(s, 1H), 7.18(s, 1H), 6.80(d, J = 2.4 Hz, 1H), 6.74(dd, J = 8.7, 2.4 Hz, 1H), 4.95(d, J = 8.4 Hz, 2H), 4.53(s, 2H), 1.82(s, 4H), 1.47(s, 18H), 1.03(d, J = 2.4 Hz, 6H), 1.01(d, J = 1.8 Hz, 6H). ^13^C NMR (75 MHz, CDCl_3_) δ 192.32, 171.96, 171.06, 165.70, 157.27, 155.18, 152.24, 144.23, 132.08, 130.64, 129.59, 121.86, 119.86, 117.04, 112.34, 110.86, 79.94, 52.13, 41.11, 28.01, 24.63, 22.61, 21.54. HRMS (ESI, *m*/*z*): Calcd for C_38_H_55_O_10_N_2_ [M + H]^+^ 699.3857, found 699.3841.

**(*E*)-4-(3-(4-((2-((tert-butoxycarbonyl)amino)-4-methylhexanoyl)oxy)-2-hydroxyphenyl)-3-oxoprop-1-en-1-yl)phenyl 2-((tert-butoxycarbonyl)amino)-4-methylhexanoate (Compound 2):** Silica gel chromatography (PE/AC = 6:1) separated to give a yellow, viscous solid, yield 79.5%. ^1^H NMR (300 MHz, CDCl_3_) δ 13.00(s,1H), 7.94(d, J = 4.2 Hz, 1H), 7.90(d, J = 10.8 Hz, 1H), 7.70(s, 1H), 7.67(s, 1H), 7.54(d, J = 15.3 Hz, 1H), 7.20(s, 1H), 7.17(s, 1H), 6.79(d, J = 2.4 Hz, 1H), 6.73(dd, J = 8.7, 2.1 Hz, 1H), 5.08(d, J = 7.5 Hz, 2H), 4.50(d, J = 4.2 Hz, 2H), 2.04(s, 2H), 1.63(s, 2H), 1.47(s, 18H), 1.42(s, 4H), 1.06(dd, J = 6.6, 2.7 Hz, 6H), 1.02–0.96(m, 6H). ^13^C NMR (75 MHz, CDCl_3_) δ 192.32, 170.55, 170.34, 164.80, 156.06, 155.32, 152.96, 145.84, 132.13, 130.68, 129.62, 121.88, 119.89, 117.81, 112.41, 110.88, 79.89, 58.52, 38.05, 28.02, 24.92, 15.42, 11.36. HRMS (ESI, *m*/*z*): Calcd for C_40_H_59_O_10_N_2_ [M + H]^+^ 727.4170, found 727.4154.

**(*E*)-4-(3-(4-(((tert-butoxycarbonyl)valyl)oxy)-2-hydroxyphenyl)-3-oxoprop-1-en-1-yl)phenyl (tert-butoxycarbonyl)valinate (Compound 3):** Silica gel chromatography (PE/AC = 8:1) separated to give a yellow, viscous solid, yield 75.3%. ^1^H NMR (300 MHz, CDCl_3_) δ 13.00 (s, 1H), 7.94(d, J = 3.6 Hz, 1H), 7.90(d, J = 11.1 Hz, 1H), 7.70(s, 1H, H-2), 7.67(s, 1H, H-6), 7.55(d, J = 15.3 Hz, 1H), 7.20(s, 1H), 7.18(s, 1H), 6.79(d, J = 2.1 Hz, 1H), 6.73(dd, J = 9.0, 2.4 Hz, 1H), 5.05(s, 2H), 4.48(s, 2H), 2.30(s, 2H), 1.47(s, 18H), 1.09(dd, J = 6.9, 2.7 Hz, 6H), 1.03(dd, J = 6.9, 3.0 Hz, 6H). ^13^C NMR (75 MHz, CDCl_3_) δ 193.14, 171.20, 170.77, 165.66, 156.90, 156.28, 152.48, 145.07, 132.97, 131.52, 129.83, 122.73, 120.72, 118.64, 113.22, 111.73, 80.73, 59.35, 31.77, 28.85, 19.68, 18.27. HRMS (ESI, *m*/*z*): Calcd for C_37_H_53_O_10_N_2_ [M + H]^+^ 685.3700, found 685.3684.

**(*E*)-2-(4-(3-(4-(((tert-butoxycarbonyl)prolyl)oxy)-2-hydroxyphenyl)-3-oxoprop-1-en-1-yl)phenyl) 1-(tert-butyl) pyrrolidine-1,2-dicarboxylate (Compound 4):** Silica gel chromatography (PE/AC = 6:1) separated to give a yellow, viscous solid, yield 78.3%. ^1^H NMR (300 MHz, CDCl_3_) δ 13.01(s, 1H), 7.94(d, J = 8.7 Hz, 1H), 7.88(s, 1H), 7.71(s, 1H), 7.66(d, J = 8.7 Hz, 1H), 7.54(d, J = 18.3Hz, 1H), 7.21(d, J = 5.4 Hz, 1H), 7.17(s, 1H), 6.79(s, 1H), 6.72(d, J = 8.7, 2.4 Hz, 1H), 4.49(d, J = 11.4 Hz, 2H), 3.56(s, 4H), 2.02(s, 8H), 1.52–1.40(s, 18H). ^13^C NMR (75 MHz, CDCl_3_) δ193.24, 171.71, 171.28, 165.68, 157.21, 155.26, 154.23, 145.68, 132.84, 131.51, 130.45, 122.76, 121.67, 120.72, 117.96, 114.26, 110.47, 80.30, 59.71, 46.62, 31.53, 28.94, 25.05, 24.25. HRMS (ESI, *m*/*z*): Calcd for C_53_H_43_O_10_N_2_ [M + H]^+^ 651.2918, found 651.2902.

**(*E*)-4-(3-(4-((N-(tert-butoxycarbonyl)-N-methylglycyl)oxy)-2-hydroxyphenyl)-3-oxoprop-1-en-1-yl)phenyl N-(tert-butoxycarbonyl)-N-methylglycinate (Compound 5):** Silica gel chromatography (PE/AC = 5:1) separated to give a yellow, viscous solid, yield 65.7%. ^1^H NMR (300 MHz, CDCl_3_) δ12.99 (s, 1H), 7.93(s, 1H), 7.88(s, 1H), 7.70(d, J = 7.2 Hz, 1H), 7.66(s, 1H), 7.55(d, J = 15.0 Hz, 1H), 7.22(d, J = 5.4 Hz, 1H), 7.19(s, 1H), 6.81(s, 1H), 6.76(s, 1H), 4.23(s, 2H), 4.15(s, 2H), 3.00(s, 6H), 1.49(s, 18H). ^13^C NMR (75 MHz, CDCl_3_) δ192.32, 167.89, 167.36, 164.87, 156.08, 154.77, 152.18, 144.23, 132.22, 130.63, 129.61, 122.68, 120.67, 118.57, 113.24, 111.66, 80.91, 50.96, 50.29, 33.65, 28.04. HRMS (ESI, *m*/*z*): Calcd for C_31_H_39_O_10_N_2_ [M + H]^+^ 599.2605, found 599.2589.

**4-((*E*)-3-(4-((S)-2-((tert-butoxycarbonyl)amino)-2-cyclopentylacetoxy)-2-hydroxyphenyl)-3-oxoprop-1-en-1-yl)phenyl(R)-2-((tert-butoxycarbonyl)amino)-2-cyclopentylacetate (Compound 6):** Silica gel chromatography (PE/AC = 8:1) separated to give a yellow, viscous solid, yield 75.3%. ^1^H NMR (300 MHz, CDCl_3_) δ 13.00(s, 1H), 7.93(d, J = 4.5 Hz, 1H), 7.89(d, J = 10.8 Hz, 1H), 7.69(s, 1H), 7.66(s, 1H), 7.54(d, J = 15.6 Hz, 1H), 7.20(s, 1H), 7.17(s, 1H), 6.79(d, J = 2.1 Hz, 1H), 6.74(dd, J = 8.7, 2.4 Hz, 1H), 5.05(s, 2H), 4.41(s, 2H), 2.36(s, 2H), 1.67(s, 16H), 1.47(s, 18H). ^13^C NMR (75 MHz, CDCl_3_) δ 192.46, 172.01, 170.56, 165.62, 157.01, 156.16, 153.03, 145.0, 133.27, 131.49, 130.42, 122.23, 119.91, 118.57, 112.90, 111.66, 80.70, 55.56, 42.88, 29.58, 29.09, 28.83, 25.87, 25.58. HRMS (ESI, *m*/*z*): Calcd for C_39_H_51_O_10_N_2_ [M + H]^+^ 707.3544, found 707.3528.

**(*E*)-4-(3-(2,4-dihydroxyphenyl)-3-oxoprop-1-en-1-yl)phenyl(R)-2-((tert-butoxycarbonyl)amino)-2-cyclopentylacetate (Compound 7):** Silica gel chromatography (PE/AC = 8:1) separated to give a yellow, viscous solid, yield 36.8%. ^1^H NMR (300 MHz, CDCl_3_) δ 13.31(s, 1H), 7.80(d, J = 15.9 Hz, 1H), 7.74(d, J = 9.6 Hz, 1H), 7.63(s, 1H), 7.60(s, 1H), 7.45(d, J = 15.9 Hz, 1H), 7.15(s, 1H), 7.12(s, 1H), 6.42(s, 1H), 6.39(s, 1H), 5.10(s, 1H), 4.41(s, 1H), 2.33(s, 1H), 1.69(s, 8H), 1.48(s, 9H). ^13^C NMR (75 MHz, CDCl_3_) δ192.06, 172.00, 166.58, 164.55, 156.53, 152.62, 143.50, 133.30, 132.38, 131.40, 130.19, 122.51, 121.09, 114.49, 109.21, 104.23, 81.18, 57.77, 42.90, 29.63, 29.15, 28.87, 25.90, 25.61. HRMS (ESI, *m*/*z*): Calcd for C_27_H_32_O_7_N [M + H]^+^ 482.2179, found 482.2163.

**4-((*E*)-3-(4-((*R*)-2-((tert-butoxycarbonyl)amino)-2-cyclohexylacetoxy)-2-hydroxyphenyl)-3-oxoprop-1-en-1-yl)phenyl(S)-2-((tert-butoxycarbonyl)amino)-2-cyclohexylacetate (Compound 8):** Silica gel chromatography (PE/AC = 8:1) separated to give a yellow, viscous solid, yield 71.4%. ^1^H NMR (300 MHz, CDCl_3_) δ 13.00(s, 1H), 7.94(d, J = 4.2 Hz, 1H), 7.90(d, J = 10.5 Hz, 1H), 7.70(s, 1H), 7.67(s, 1H), 7.54(d, J = 15.6 Hz, 1H), 7.21(s, 1H), 7.18(s, 1H), 6.79(d, J = 2.4 Hz, 1H), 6.74(d, J = 6.3 Hz, 1H), 5.06(s, 2H), 4.42(s, 2H), 1.80(s, 8H), 1.47(s, 18H), 1.42(s, 2H), 1.25(s, 12H). ^13^C NMR (75 MHz, CDCl_3_) δ193.11, 171.88, 170.82, 165.60, 157.37, 156.18, 152.48, 145.03, 132.91, 131.47, 130.41, 122.17, 120.68, 118.59, 113.99, 111.72, 80.17, 55.95, 40.25, 30.08, 28.83, 27.41, 26.49. HRMS (ESI, *m*/*z*): Calcd for C_41_H_55_O_10_N_2_ [M + H]^+^ 735.3857, found 735.3841.

**(*E*)-4-(3-(2,4-dihydroxyphenyl)-3-oxoprop-1-en-1-yl)phenyl(S)-2-((tert-butoxycarbonyl)amino)-2-cyclohexylacetate (Compound 9):** Silica gel chromatography (PE/AC = 8:1) separated to give a yellow, viscous solid, yield 42.7%. ^1^H NMR (300 MHz, CDCl_3_) δ13.30(s, 1H), 7.86(d, J = 15.0 Hz, 1H), 7.72(s, 1H), 7.62(s, 1H), 7.54(s, 1H), 7.42(s, 1H), 7.13(d, J = 6.9 Hz, 1H), 6.87(s, 1H), 6.71(d, J = 24.6 Hz, 1H), 6.39(s, 1H), 5.09(s, 1H), 4.42(s, 1H), 1.88(s, 1H), 1.80(s, 4H), 1.48(s, 9H), 1.27(s, 6H). ^13^C NMR (75 MHz, CDCl_3_) δ191.30, 170.78, 166.30, 163.51, 156.32, 151.81, 142.45, 132.54, 131.64, 130.66, 129.44, 121.79, 119.83, 113.86, 107.87, 103.56, 83.27, 59.01, 41.11, 29.39, 28.15, 25.36. HRMS (ESI, *m*/*z*): Calcd for C_28_H_34_O_7_N [M + H]^+^ 496.2335, found 496.2319.

**4-((*E*)-3-(4-(((tert-butoxycarbonyl)-d-alanyl)oxy)phenyl)acryloyl)-3-hydroxyphenyl (tert-butoxycarbonyl)-L-alaninate (Compound 10):** Silica gel chromatography (PE/AC = 6:1) separated to give a yellow, viscous solid, yield 62.5%. ^1^H NMR (300 MHz, CDCl_3_) δ 12.99(s, 1H), 7.93(d, J = 5.4 Hz, 1H), 7.89(d, J = 11.7 Hz, 1H), 7.70(s, 1H), 7.67(s, 1H), 7.54(d, J = 15.6 Hz, 1H), 7.21(s, 1H), 7.18(s, 1H), 6.80(d, J = 2.4 Hz, 1H), 6.74(dd, J = 9.0, 2.4 Hz, 1H), 5.08(s, 2H), 4.55(s, 2H), 1.58–1.54(m, 6H), 1.47(s, 18H). ^13^C NMR (75 MHz, CDCl_3_) δ 192.41, 171.42, 170.84, 164.93, 155.85, 152.29, 154.74, 144.31, 132.22, 130.76, 129.72, 121.89, 119.99, 117.89, 112.34, 110.95, 80.05, 54.22, 27.22, 14.19. HRMS (ESI, *m*/*z*): Calcd for C_31_H_39_O_10_N_2_ [M + H]^+^ 598.2526, found 598.2510.

**(*E*)-4-(3-(2,4-dihydroxyphenyl)-3-oxoprop-1-en-1-yl)phenyl(tert-butoxycarbonyl)-D-alaninate (Compound 11):** Silica gel chromatography (PE/AC = 6:1) separated to give a yellow, viscous solid, yield 54.4%. ^1^H NMR (300 MHz, CDCl_3_) δ 13.30(s, 1H), 7.81(d, J = 15.0 Hz, 1H), 7.75(d, J = 9.3 Hz, 1H), 7.63(s, 1H), 7.61(s, 1H), 7.46(d, J = 15.0 Hz, 1H), 7.15(s, 1H), 7.13(s, 1H), 6.41(s, 1H), 6.40(d, J = 3.6 Hz, 1H), 5.09(s, 1H), 4.55(s, 1H), 1.58(s, 3H), 1.48(s, 9H). ^13^C NMR (75 MHz, CDCl_3_) δ 191.22, 171.02, 165.43, 163.51, 154.62, 151.78, 142.70, 132.47, 131.58, 129.35, 121.59, 120.26, 113.75, 108.27, 103.42, 81.96, 50.74, 29.72, 17.88. HRMS (ESI, *m*/*z*): Calcd for C_23_H_26_O_7_N [M + H]^+^ 428.1709, found 428.1693.

**4-((*E*)-3-(4-((2-((tert-butoxycarbonyl)amino)-4-phenylbutanoyl)oxy)-2-hydroxyphenyl)-3-oxoprop-1-en-1-yl)phenyl(2S)-2-((tert-butoxycarbonyl)amino)-4-phenylbutanoate (Compound 12):** Silica gel chromatography (PE/AC = 5:1) separated to give a yellow, viscous solid, yield 60.7%. ^1^H NMR (300 MHz, CDCl_3_) δ 13.00(s, 1H), 7.94(s, 1H), 7.89(d, J = 9.6 Hz, 1H), 7.69(s, 1H), 7.66(s, 1H), 7.54(d, J = 15.6 Hz, 1H), 7.31(d, J = 7.6 Hz, 4H), 7.24(s, 4H), 7.21(s, 2H), 7.17(s, 1H), 7.14(s, 1H), 6.77(d, J = 2.4 Hz, 1H), 6.70(dd, J = 8.7, 2.4 Hz, 1H), 5.13(d, J = 8.4 Hz, 2H), 4.57(s, 2H), 2.80(q, J = 4.8 Hz, 4H), 2.31(s, 2H), 2.13(s, 2H), 1.48(s, 18H). ^13^C NMR (75 MHz, CDCl_3_) δ 193.13, 171.67, 171.08, 165.68, 156.90, 155.94, 152.95, 144.34, 140.96, 133.01, 131.51, 130.46, 129.19, 128.99, 126.33, 122.65, 120.78, 118.66, 113.10, 111.72, 80.63, 54.12, 34.67, 32.33, 28.88. HRMS (ESI, *m*/*z*): Calcd for C_45_H_51_O_10_N_2_ [M + H]^+^ 779.3544, found 779.3528.

**(*E*)-4-(3-(2,4-dihydroxyphenyl)-3-oxoprop-1-en-1-yl)phenyl(S)-2-((tert-butoxycarbonyl)amino)-4-phenylbutanoate (Compound 13):** Silica gel chromatography (PE/AC = 5:1) separated to give a yellow, viscous solid, yield 40.2%. ^1^H NMR (300 MHz, CDCl_3_) δ 13.31(s, 1H), 7.80(d, J = 15.6 Hz, 1H), 7.72(d, J = 9.9 Hz, 1H), 7.61(s, 1H), 7.58(s, 1H), 7.44(d, J = 15.6 Hz, 1H), 7.32(s, 1H), 7.30(s, 1H), 7.24(s, 2H), 7.22(s, 1H), 7.11(s, 1H), 7.08(s, 1H), 6.39(s, 2H), 5.18(s, 1H), 4.56(s, 1H), 2.81(s, 2H), 2.15(s, 2H), 1.49(s, 9H). ^13^C NMR (75 MHz, CDCl_3_) δ 192.59, 170.45, 166.19, 163.51, 155.60, 152.07, 142.44, 140.39, 132.92, 131.99, 129.77, 128.78, 128.55, 126.93, 122.00, 120.03, 113.64, 108.26, 102.81, 83.18, 53.76, 34.08, 31.93, 28.45. HRMS (ESI, *m*/*z*): Calcd for C_30_H_32_O_7_N [M + H]^+^ 518.2179, found 518.2163.

**4-((*E*)-3-(4-(((S)-3-(2-bromophenyl)-2-((tert-butoxycarbonyl)amino)propanoyl)oxy)-2-hydroxyphenyl)-3-oxoprop-1-en-1-yl)phenyl(R)-3-(2-bromophenyl)-2-((tert-butoxycarbonyl)amino)propanoate (Compound 14):** Silica gel chromatography (PE/AC = 5:1) separated to give a yellow, viscous solid, yield 57.1%. ^1^H NMR (300 MHz, CDCl_3_) δ 12.98(s, 1H), 7.92(s, 1H), 7.88(d, J = 8.1 Hz, 1H), 7.68(s, 1H), 7.65(s, 1H), 7.62(s, 1H), 7.59(s, 1H), 7.54(d, J = 15.6 Hz, 1H), 7.30(s, 2H), 7.28(s, 2H), 7.17(s, 2H), 7.13(s, 1H), 7.10(s, 1H), 6.71(s, 1H), 6.67(dd, J = 8.7, 2.1 Hz, 1H), 5.14(s, 2H), 4.86(s, 2H), 3.42(s, 2H), 3.30(s, 2H), 1.41(s, 18H). ^13^C NMR (75 MHz, CDCl_3_) δ 193.13, 170.92, 170.53, 165.63, 157.05, 155.91, 153.11, 145.05, 135.89, 133.70, 133.15, 132.05, 131.43, 130.41, 129.53, 128.23, 125.59, 122.63, 120.28, 118.65, 113.26, 111.66, 80.84, 54.08, 39.01, 28.28. HRMS (ESI, *m*/*z*): Calcd for C_43_H_45_O_10_Br_2_N_2_ [M + H]^+^ 907.1441, found 907.1425.

**(*E*)-4-(3-(2,4-dihydroxyphenyl)-3-oxoprop-1-en-1-yl)phenyl(R)-3-(2-bromophenyl)-2-((tert-butoxycarbonyl)amino)propanoate (Compound 15):** Silica gel chromatography (PE/AC = 5:1) separated to give a yellow, viscous solid, yield 45.5%. ^1^H NMR (300 MHz, CDCl_3_) δ13.31(s, 1H), 7.80(d, J = 15.6 Hz, 1H), 7.74(d, J = 9.6 Hz, 1H), 7.61(s, 2H), 7.59(s, 1H), 7.45(d, J = 15.3 Hz, 1H), 7.30(s, 1H), 7.29(s, 1H), 7.18(s, 1H), 7.08(s, 1H), 7.05(s, 1H), 6.40(s, 2H), 5.19(s, 1H), 4.88(s, 1H), 3.42(s, 2H), 1.42(s, 9H). ^13^C NMR (75 MHz, CDCl_3_) δ 191.20, 170.21, 166.13, 164.16, 154.50, 151.58, 142.21, 135.25, 132.84, 132.48, 131.54, 131.17, 129.31, 128.11, 127.41, 123.90, 121.58, 119.37, 112.60, 107.02, 103.88, 80.41, 51.90, 36.75, 27.01. HRMS (ESI, *m*/*z*): Calcd for C_29_H_29_O_7_BrN [M + H]^+^ 582.1127, found 582.1111.

**4-((*E*)-3-(4-(((S)-2-((tert-butoxycarbonyl)amino)-3-(2-chlorophenyl)propanoyl)oxy)-2-hydroxyphenyl)-3-oxoprop-1-en-1-yl)phenyl(R)-2-((tert-butoxycarbonyl)amino)-3-(2-chlorophenyl)propanoate (Compound 16):** Silica gel chromatography (PE/AC = 5:1) separated to give a yellow, viscous solid, yield 54.9%. ^1^H NMR (300 MHz, CDCl_3_) δ 12.98(s, 1H), 7.92(s, 1H), 7.88(d, J = 8.4 Hz, 1H), 7.68(s, 1H), 7.65(s, 1H), 7.62(s, 1H), 7.59(s, 1H), 7.54(d, J = 15.6 Hz, 1H), 7.30(s, 2H), 7.28(s, 2H), 7.18(s, 2H), 7.13(s, 1H), 7.10(s, 1H), 6.71(d, J = 2.1, 1H), 6.67(dd, J = 8.7, 2.4 Hz, 1H), 5.09(s, 2H), 4.86(s, 2H), 3.40(s, 2H), 3.26(s, 2H), 1.41(s, 18H). ^13^C NMR (75 MHz, CDCl_3_) δ 192.71, 170.62, 170.04, 165.19, 156.64, 155.39, 152.65, 145.29, 134.70, 134.03, 133.15, 132.05, 131.43, 130.41, 129.53, 128.23, 125.59, 122.17, 120.31, 118.21, 113.17, 111.23, 80.34, 53.55, 36.09, 28.36. HRMS (ESI, *m*/*z*): Calcd for C_43_H_45_O_10_Cl_2_N_2_ [M + H]^+^ 820.2134, found 820.2118.

**(*E*)-4-(3-(2,4-dihydroxyphenyl)-3-oxoprop-1-en-1-yl)phenyl(R)-2-((tert-butoxycarbonyl)amino)-3-(2-chlorophenyl)propanoate (Compound 17):** Silica gel chromatography (PE/AC = 5:1) separated to give a yellow, viscous solid, yield 43.6%. ^1^H NMR (300 MHz, CDCl_3_) δ 13.31(s,1H), 7.80(d, J = 15.3 Hz, 1H), 7.75(d, J = 9.0 Hz, 1H), 7.62(s, 1H), 7.59(s, 1H), 7.46(d, J = 16.2 Hz, 1H), 7.40(s, 1H), 7.28–7.27(m, 1H), 7.24(d, J = 8.4 Hz, 2H), 7.08(s, 1H), 7.05(s, 1H), 6.40(s, 2H), 5.19(s, 1H), 4.85(s, 1H), 3.45(s, 2H), 1.42(s, 9H). ^13^C NMR (75 MHz, CDCl_3_) δ 191.36, 171.20, 167.03, 164.24, 155.77, 152.94, 144.07, 135.13, 134.31, 133.34, 132.42, 132.07, 130.40, 130.17, 129.42, 127.67, 122.41, 120.75, 114.64, 108.15, 103.61, 81.37, 54.45, 37.00, 26.32. HRMS (ESI, *m*/*z*): Calcd for C_29_H_29_O_7_ClN [M + H]^+^ 538.6474, found 538.6458.

**4-((*E*)-3-(4-((S)-2-((tert-butoxycarbonyl)amino)-2-phenylacetoxy)-2-hydroxyphenyl)-3-oxoprop-1-en-1-yl)phenyl(R)-2-((tert-butoxycarbonyl)amino)-2-phenylacetate (Compound 18):** Silica gel chromatography (PE/AC = 5:1) separated to give a yellow, viscous solid, yield 60.7%. ^1^H NMR (300 MHz, CDCl_3_) δ 12.95(s, 1H), 7.88(s, 1H), 7.84(d, J = 6.6 Hz, 1H), 7.64(s, 1H), 7.61(s, 1H), 7.51(s, 1H), 7.46(s, 5H), 7.41(d, J = 5.7 Hz, 5H), 7.11(s, 1H, H-5), 7.08(s, 1H), 6.71–6.62(m, 2H), 5.53(s, 4H), 1.46(s, 18H). ^13^C NMR (75 MHz, CDCl_3_) δ193.07, 169.57, 169.10, 164.73, 155.76, 154.61, 144.18, 134.05, 132.16, 130.61, 129.54, 128.94, 128.66, 127.05, 121.68, 119.87, 117.00, 112.17, 110.42, 79.36, 57.01, 28.00. HRMS (ESI, *m*/*z*): Calcd for C_41_H_43_O_10_N_2_ [M + H]^+^ 723.2918, found 723.2902.

**(*E*)-4-(3-(2,4-dihydroxyphenyl)-3-oxoprop-1-en-1-yl)phenyl(R)-2-((tert-butoxycarbonyl)amino)-2-phenylacetate (Compound 19):** Silica gel chromatography (PE/AC = 5:1) separated to give a yellow, viscous solid, yield 42.5%. ^1^H NMR (300 MHz, CDCl_3_) δ 13.30(s, 1H), 7.78(d, J = 14.7 Hz, 1H), 7.72(d, J = 9.6 Hz, 1H), 7.58(s, 1H), 7.55(s, 1H), 7.50(s, 1H), 7.46(d, J = 3.9 Hz, 2H), 7.43(s, 1H), 7.41(s, 2H), 7.06(s, 1H), 7.03(s, 1H), 6.38(s, 2H), 5.30(s, 2H), 1.47(s, 9H). ^13^C NMR (75 MHz, CDCl_3_) δ192.94, 170.39, 167.01, 164.28, 156.31, 152.47, 143.07, 135.47, 133.20, 132.38, 131.41, 130.15, 129.86, 129.64, 127.93, 121.89, 121.10, 118.35, 116.70, 114.59, 109.87, 104.77, 81.89, 53.71, 28.87. HRMS (ESI, *m*/*z*): Calcd for C_28_H_28_O_7_N [M + H]^+^ 490.1866, found 490.1850.

**4-((*E*)-3-(4-(((tert-butoxycarbonyl)-D-phenylalanyl)oxy)phenyl)acryloyl)-3-hydroxyphenyl (tert-butoxycarbonyl)-L-phenylalaninate (Compound 20):** Silica gel chromatography (PE/AC = 6:1) separated to give a yellow, viscous solid, yield 63.5%. ^1^H NMR (300 MHz, CDCl_3_) δ 12.99(s, 1H), 7.92(s, 1H), 7.88(d, J = 7.2 Hz, 1H), 7.68(s,1H), 7.65(s, 1H), 7.53(d, J = 15.6 Hz, 1H), 7.36(s, 2H), 7.34(s, 4H), 7.24(s, 4H), 7.08(s, 1H), 7.06(s, 1H), 6.69(s, 1H), 6.62(dd, J = 8.7, 2.1 Hz, 1H), 5.06(s, 2H), 4.78(s, 2H), 3.24(s, 4H), 1.45(s, 18H). ^13^C NMR (75 MHz, CDCl_3_) δ193.13, 171.23, 170.62, 165.62, 156.77, 155.90, 152.83, 143.77, 136.41, 132.97, 131.43, 130.41, 129.94, 129.34, 127.93, 122.62, 120.37, 117.95, 113.09, 111.20, 80.90, 54.08, 38.81, 27.70. HRMS (ESI, *m*/*z*): Calcd for C_43_H_47_O_10_N [M + H]^+^ 751.3231, found 751.3215.

**(*E*)-4-(3-(2,4-dihydroxyphenyl)-3-oxoprop-1-en-1-yl)phenyl(tert-butoxycarbonyl)-D-phenylalaninate (Compound 21):** Silica gel chromatography (PE/AC = 6:1) separated to give a yellow, viscous solid, yield 40.1%. ^1^H NMR (300 MHz, CDCl_3_) δ 13.31(s, 1H), 7.79(d, J = 15.3 Hz, 1H), 7.73(d, J = 9.3 Hz, 1H), 7.61(s, 1H), 7.57(s, 1H), 7.44(d, J = 15.6 Hz, 1H), 7.36(s, 1H), 7.33(d, J = 5.1 Hz, 2H), 7.23(s, 2H), 7.03(s, 1H), 7.00(s, 1H), 6.41(s, 2H), 5.10(s, 1H), 4.81(d, J = 7.5 Hz, 1H), 3.23(s, 2H), 1.46(s, 9H). ^13^C NMR (75 MHz, CDCl_3_) δ192.06, 170.80, 166.47, 164.34, 156.22, 152.40, 143.54, 137.57, 133.33, 132.41, 130.17, 129.93, 129.41, 128.00, 123.11, 120.44, 114.17, 109.62, 104.33, 81.10, 53.70, 37.92, 25.05. HRMS (ESI, *m*/*z*): Calcd for C_29_H_30_O_7_N [M + H]^+^ 504.2022, found 504.2006.

**4-((*E*)-3-(4-(((S)-2-((tert-butoxycarbonyl)amino)-3-(p-tolyl)propanoyl)oxy)-2-hydroxyphenyl)-3-oxoprop-1-en-1-yl)phenyl(R)-2-((tert-butoxycarbonyl)amino)-3-(p-tolyl)propanoate (Compound 22):** Silica gel chromatography (PE/AC = 6:1) separated to give a yellow, viscous solid, yield 67.0%. ^1^H NMR (300 MHz, CDCl_3_) δ 12.99(s, 1H), 7.93(s, 1H), 7.88(d, J = 7.2 Hz, 1H), 7.68(s, 1H), 7.65(s, 1H), 7.54(d, J = 15.3 Hz, 1H), 7.19–7.11(m, 8H), 7.10(s, 1H), 7.08(s, 1H), 6.70(d, J = 2.1 Hz, 1H), 6.65(dd, J = 9.0, 2.4 Hz, 1H), 5.03(s, 2H), 4.74(s, 2H), 3.20(s, 4H), 2.35(s, 6H), 1.45(s, 18H). ^13^C NMR (75 MHz, CDCl_3_) δ191.80, 170.76, 170.36, 165.21, 156.40, 155.30, 152.47, 144.62, 137.13, 132.54, 131.02, 130.00, 129.61, 129.38, 122.24, 119.85, 117.69, 112.72, 111.05, 80.44, 54.91, 37.88, 28.41, 21.72. HRMS (ESI, *m*/*z*): Calcd for C_45_H_51_O_10_N_2_ [M + H]^+^ 779.3544, found 779.3528.

**(*E*)-4-(3-(2,4-dihydroxyphenyl)-3-oxoprop-1-en-1-yl)phenyl(R)-2-((tert-butoxycarbonyl)amino)-3-(p-tolyl)propanoate (Compound 23):** Silica gel chromatography (PE/AC = 6:1) separated to give a yellow, viscous solid, yield 42.5%. ^1^H NMR (300 MHz, CDCl_3_) δ 13.31(s, 1H), 7.80(d, J = 15.6 Hz, 1H), 7.73(d, J = 9.6 Hz, 1H), 7.61(s, 1H), 7.58(s, 1H), 7.45(d, J = 15.3 Hz, 1H), 7.16(d, J = 8.4 Hz, 2H), 7.14–7.10(m, 2H), 7.06(s, 1H), 7.03(s, 1H), 6.39(s, 2H), 5.07(s, 1H), 4.77(s, 1H), 3.20(d, J = 6.0 Hz, 2H), 2.35(s, 3H), 1.45(s, 9H). ^13^C NMR (75 MHz, CDCl_3_) δ191.21, 170.20, 166.17, 163.67, 155.14, 151.73, 143.21, 136.45, 132.46, 131.89, 131.36, 129.25, 128.94, 121.58, 120.20, 115.36, 113.75, 103.44, 80.61, 54.35, 37.38, 28.49, 19.09. HRMS (ESI, *m*/*z*): Calcd for C_30_H_32_O_7_N [M + H]^+^ 486.9753, found 486.9737.

### 3.3. Cytotoxic Activity Assay

5-fluorouracil (5-FU, Beijing Solarbio Technology Co., Ltd., Beijing, China) was selected as the positive control drug, and the inhibitory effects of the synthesized isoliquiritigenin derivatives on the Bel-7402 (Wuhan Punuosai Life Science and Technology Co., Ltd., Wuhan, China), A549 (Cell Bank, Chinese Academy of Medical Sciences, Shanghai, China), Hela (Chinese RSBM Public Benefit Cell Bank, Taiyuan, China), MCF-7 (Chinese RSBM Public Benefit Cell Bank, Taiyuan, China), PC-3M (Cell Bank, Chinese Academy of Medical Sciences, Shanghai, China), and HUCEC (Wuhan Punuosai Life Science and Technology Co., Ltd., Wuhan, China) cell lines were evaluated using the MTT method. MCF-7, Hela, and HUCEC were cultured in DMEM (Beijing Solarbio) containing 10% FBS, 100 U/mL penicillin, and 100 mg/mL streptomycin at 37 °C and 5% CO_2_. Bel-7402, A549, PC-3M were cultured in RPMI-1640 (Beijing Solarbio) media supplemented with 10% FBS (Wuhan Punuosai Life Science and Technology Co., Ltd., Wuhan, China), 100 U/mL penicillin, and 100 mg/mL streptomycin at 37 °C and 5% CO_2_. MTT (Beijing Solarbio) was prepared with PBS at a concentration of 5 mg/mL. All tested drugs were prepared in a solution with a concentration of 20 mM, with dimethyl sulfoxide as the solvent.

The cells were inoculated into 96-well plates at a density of 5 × 10^3^ cells/well. When the cell fusion reached 70–80%, the drug was treated for 48 h. Subsequently, 20 μL MTT was added to each well and incubated at 37 °C with 5% CO_2_ for 4 h. After that, the medium was discarded and 150 μL DMSO was added to each well. The absorbance was measured at 490 nm with an enzymoleter.

### 3.4. Cell Scratch Assay

Hela cells were inoculated into the 6-well plate at a density of 1 × 10^4^ cells/well. When the cell growth reached 80–90% density, the medium was discarded, and a straight line was drawn with the gun head perpendicular to the horizontal line behind the 6-well plate. PBS was used to wash off the delimit cells twice; then, different concentrations of drugs were added to the PBS, and cell migration images of 0 h were recorded under the microscope. It was put into the incubator for 48 h and then photographed again, and the cell migration images were recorded at the same position for 48 h.

### 3.5. Cell Clonal Formation Assay

Hela cells were inoculated into the 6-well plate at a density of 1 × 10^3^ cells/well. After the cells were attached to the wall and treated with drugs for 48 h, the medium was discarded and the complete medium was added, and the liquid was changed every 4 days. After 8–9 days of culture, visible cell masses in the culture plate were observed, that is, cell cloning occurred. After clone formation, we discarded the medium, added PBS to clean it 3 times, and then added 4% paraformaldehyde along the hole to fix for 15 min. After discarding the paraformaldehyde, it was washed with PBS for 3 times, and then we added 0.1% crystal violet dye along the hole for 15 min. After dyeing, the crystal violet was recovered, washed with PBS 3–4 times, dried at room temperature, and photographed.

### 3.6. Cell DAPI Staining Assay

Hela cells were seeded in 6-well plates at a density of 1 × 10^4^/well, and the cells were treated with drugs for 48 h after the cells grew to 80% density, the medium was discarded, PBS was added to wash 3 times, and 4% paraformaldehyde was added along the wells for 15 min. The paraformaldehyde was discarded, PBS was added for washing 3 times, DAPI staining solution was added along the well to protect from light for 15 min. Then, the staining solution was washed off with PBS, and we immediately took pictures under the microscope to observe the morphological changes.

### 3.7. Cell Apoptosis Assay

Hela cells were seeded in 6-well plates at a density of 1 × 10^5^/well and cultured in a cell culture incubator for 24 h. The medium was discarded, and PBS was added for 3 washes. Drug treatment lasted 48 h, the medium was discarded, PBS was added for 3 washes, trypsin digestion without EDTA was added, 500 μL of binding buffer was added to each group and gently blown into a single-cell suspension, 5 μL of Annexin V-FITC was mixed, and 5 μL of propidium was added to iodide. We set up a single positive tube and a blank tube at the same time, mixed the sample well at room temperature, protected it from light, reacted it for 10 min, and then observed and detected the flow cytometer within 1 h.

### 3.8. Molecular Docking

The X-ray crystal structures of PI3K (PDB ID: 5GJI) and mTOR (PDB ID: 7SOQ) were obtained from the Protein Data Bank (https://www.rcsb.org/ accessed on 2 March 2024). The structure of isoliquiritigenin was downloaded from the PubChem database (https://www.pubchem.ncbi.nlm.nih.gov/ accessed on 2 March 2024), and the structure of compound 9 was drawn from Chemdraw V20.0. We used ChemBio3D Ultra 14.0 software (PerkinElmer Informatics) for optimization. Auto Dock Vina 1.2.0 software (Center for Computational Structural Biology) was used to dock conformation. PyMOL 2.5.2 was used to visualize the conformation.

### 3.9. RT-qPCR

After treating Hela cells with different concentrations of drugs for 48 h, the total RNA was extracted according to the instructions of the RNA extraction kit, and the RNA concentration and purity were determined using an ultra-micro spectrophotometer. The A260-A280 ratio was between 1.8 and 2.1 for reverse transcription to synthesize the cDNA template. The primer sequences (designed and synthesized by Sangon Biotech) are shown in Table 2, and the expression levels of the target gene in the blank control group were used as the reference factor. The 2^−△△Ct^ method was used for analysis, and the result was calculated as follows: relative expression amount = 2^−(Ct target gene-Ct internal reference gene)^.

## 4. Conclusions

In this study, a series of isoliquiritigenin derivatives was designed and synthesized. The anti-tumor activity of Bel-7402, A549, Hela, MCF-7, and PC-3M was evaluated via the MTT method using 5-FU and Isoliquiritigenin as positive control drugs. The results showed that compound 9, containing the macromolecular amino acid cyclohexyl glycine, had strong inhibitory effects on all five types of tumor cells. The experiments showed that compound 9 induced Hela cell apoptosis and autophagy through the PI3K/Akt/mTOR pathway in a concentration-dependent manner.

## Figures and Tables

**Table 1:** Inhibition rates of target compounds on proliferation of different tumor cells.

Compound	IC50 (μmoL/L)				
	Bel-7402	A549	Hela	MCF-7	PC-3M
ISL	160	122.9	126.5	133.7	141.7
5-FU	40.31	53.28	33.59	80.66	15.07
**1**	>200	>200	>200	>200	>200
**2**	>200	>200	>200	>200	>200
**3**	>200	>200	>200	>200	>200
**4**	>200	>200	>200	>200	>200
**5**	>200	>200	>200	>200	>200
**6**	>200	>200	>200	>200	>200
**7**	27.91	27.39	28.7	29.8	33.2
**8**	>200	>200	>200	>200	>200
**9**	27.85	23.68	14.36	21.24	27.01
**10**	>200	>200	>200	>200	>200
**11**	87.6	100.42	99.9	159.6	121.8
**12**	>200	>200	>200	>200	>200
**13**	35.3	48.9	29.2	60.5	37.4
**14**	>200	>200	>200	>200	>200
**15**	38.4	42.57	27.36	50.9	30.2
**16**	>200	>200	>200	>200	>200
**17**	33.18	40.69	30.4	60.2	38.1
**18**	>200	>200	>200	>200	>200
**19**	66.9	93.8	74.9	152.9	108.4
**20**	>200	>200	>200	>200	>200
**21**	45.3	60.2	66.5	75.7	87.5
**22**	>200	>200	>200	>200	>200
**23**	63.5	80.9	72.4	130.5	83.6

**Table 2:** Primer sequences.

The Name of the Gene	Sequence
GAPDH	Forward: 5′-TGACATCAAGAAGGTGGTGAAGCAG-3′ Forward: 5′-GTGTCGCTGTTGAAGTCAGAGGAG-3′
PI3K	Forward: 5′-CGGTGACTGTGTGGGACTTATTGAG-3′ Reverse: 5′-TGTAGTGTGTGGCTGTTGAACTGC-3′
AKT	Forward: 5′-GCAGGATGTGGACCAACGTGAG-3′ Reverse: 5′-GCAGGCAGCGGATGATGAAGG-3′
mTOR	Forward: 5′-GTGTTGCAGAGACTTGATGGAGGAG-3′ Reverse: 5′-CTGTGAAGGCAGAAGGTCGGAATG-3′
Beclin1	Forward: 5′-ACATCTGGCACAGTGGACAGTTTG-3′ Reverse: 5′-AGCATGGAGCAGCAACACAGTC-3′
LC3A	Forward: 5′-GCCTTCTTCCTGCTGGTGAACC-3′ Reverse: 5′-TCCTCGTCTTTCTCCTGCTCGTAG-3′
Bcl-2	Forward: 5′-TACGAGTGGGATGCGGGAGATG-3′ Reverse: 5′-CCGGGCTGGGAGGAGAAGATG-3′
Bad	Forward: 5′-GCCAACCAGCAGCAGCCATC-3′ Reverse: 5′-CCCCATCCCTTCGTCGTCCTC-3′
Caspase-3	Forward: 5′-GTGGAGGCCGACTTCTTGTATGC-3′ Reverse: 5′-TGGCACAAAGCGACTGGATGAAC-3′
P70S6K	Forward: 5′-ACACCTTCTGCGGCACCATTG-3′ Reverse: 5′-CCCAGGCTCCACCAGTCCAC-3′

## Data Availability

The original contributions presented in the study are included in the article/supplementary material, further inquiries can be directed to the corresponding authors.
